# Accuracy of multiparametric magnetic resonance imaging for diagnosing prostate Cancer: a systematic review and meta-analysis

**DOI:** 10.1186/s12885-019-6434-2

**Published:** 2019-12-23

**Authors:** Liang Zhen, Xiaoqiang Liu, Chen Yegang, Yang Yongjiao, Xu Yawei, Kang Jiaqi, Wang Xianhao, Song Yuxuan, Hu Rui, Zhang Wei, Ou Ningjing

**Affiliations:** 1 Department of Urology, Tianjin Medical University General Hospital, Tianjin, 300211 People’s Republic of China; 2 Department of Urology, Second Hospital of Tianjin Medical University, Tianjin, China

## Abstract

**Background:**

The application of multiparametric magnetic resonance imaging (mpMRI) for diagnosis of prostate cancer has been recommended by the European Association of Urology (EAU), National Comprehensive Cancer Network (NCCN), and European Society of Urogenital Radiology (ESUR) guidelines. The purpose of this study is to systematically review the literature on assessing the accuracy of mpMRI in patients with suspicion of prostate cancer.

**Method:**

We searched Embase, Pubmed and Cochrane online databases from January 12,000 to October 272,018 to extract articles exploring the possibilities that the pre-biopsy mpMRI can enhance the diagnosis accuracy of prostate cancer. The numbers of true- and false-negative results and true- and false-positive ones were extracted to calculate the corresponding sensitivity and specificity of mpMRI. Study quality was assessed using QUADAS-2 tool. Random effects meta-analysis and a hierarchical summary receiver operating characteristic (HSROC) plot were performed for further study.

**Results:**

After searching, we acquired 3741 articles for reference, of which 29 studies with 8503 participants were eligible for inclusion. MpMRI maintained impressive diagnostic value, the area under the HSROC curve was 0.87 (95%CI,0.84–0.90). The sensitivity and specificity for mpMRI were 0.87 [95%CI, 0.81–0.91] and 0.68 [95%CI,0.56–0.79] respectively. The positive likelihood ratio was 2.73 [95%CI 1.90–3.90]; negative likelihood ratio was 0.19 [95% CI 0.14,-0.27]. The risk of publication bias was negligible with *P* = 0.96.

**Conclusion:**

Results of the meta-analysis suggest that mpMRI is a sensitive tool to diagnose prostate cancer. However, because of the high heterogeneity existing among the included studies, further studies are needed to apply the results of this meta-analysis in clinic.

## Background

Prostate cancer (PCa) is the most commonly diagnosed disease for male around the world [1]. The patients diagnosed by 2012 were 1.1 million, which accounted for 15% of the malignancy tumor, and its incidence and mortality have been increasing [2]. The current standard of diagnosing PCa in suspicious men depends on transrectal ultrasound (TRUS)-guided prostate biopsy test which contains: transrectal ultrasound guided systematic biopsy; transperineal template biopsy and other method guided by transrectal ultrasound without knowing the exact location of cancer. According to a recent prospective study, the sensitivity of TRUS-biopsy to diagnose PCa is only 70.4% [3]. With the improvement in technology and the progress of modern medicine, patients have higher expectations for the prognosis. Multiparametric magnetic resonance imaging (mpMRI) has been increasingly used for guiding several aspects of prostate cancer management, including detection, staging, and treatment. The established parameters of mpMRI included T2-weighted images (T2WI), diffusion-weighted imaging (DWI), dynamic contrast-enhancement (DCE), and MR spectroscopy [4]. Abundant evidence based literature has shown that the pre-biopsy mpMRI can be used to improve the diagnosis accuracy of prostate cancer. MRI can provide internal zonal anatomy of the prostate and its extraprostatic extension of tumor, which can improve functional assessment and tumor grading in clinic [5]. The increasingly better resolution with the best depiction of prostate contours could also facilitate tumor localization [6]. In a addition, Yerram et al. [7] indicated that a low suspicion lesions on mpMRI has been shown to have a reliable negative predictive value (NPV) (90–98%) for either low-grade tumors or negative biopsies that are suitable for active surveillance. Two systematic meta-analyses which explored the role of mpMRI in PCa have been published recently. In the study by Hamoen et al. [8] which evaluated 14 studies, its pooled sensitivity and specificity were 0.81 (95% CI 0.76–0.85) and 0.77 (95% CI 0.68–0.86), respectively. In a more recent meta-analysis by Moldovan et al. [9] the pooled NPV for overall prostate cancer was 82.4% (interquartile range IQR, 69.0–92.4%) and 88.1% (IQR, 85.7–92.3) for clinical significant prostate cancer (csPCa) respectively.

However results of mpMRI may vary sharply because of difficulties in interpretation, lacking of standardized criteria for positive definition and the ability of radiologists [10]. Besides, the disadvantages of mpMRI such as equipment-specialization and time-consuming also impede its wide application [11]. Therefore, the routine application of mpMRI is still a topic of controversy due to the high variability among studies evaluating the diagnostic accuracy of mpMRI in staging and prediction of prostate cancer [12].

Some authors believed that mpMRI could be used as a preliminary screening so that some unnecessary prostate biopsies could be avoided even for the patients in active surveilance [13]. However, evidences to support such viewpoint are not convincing enough and it depends upon the sensitivity and specificity of mpMRI [14, 15] which would vary due to different MRI protocol, standard reference, and study quality. Ivo et al. [14, 15] indicated that whether patients with negative mpMRI findings could obviate biopsy remained highly controversial and it was still premature to draw a definite conclusion. Aydin et al. [16] indicated both highly vascularized benign prostatic hyperplasia nodules and prostatitis could lead to increased vessel enhancement in DCE imaging, which may cause low specificity of mpMRI. The selection bias of the two previous meta-analysis were of high level of heterogeneity because they did not exclude retrospectively designed study, besides, subgroup analysis was not performed specifically. Therefore the purpose of this study is to perform a systematic review and meta-analysis to estimate the diagnostic performance of mpMRI for detecting prostate cancer.

## Method

A systematic review was conducted under the guidance of the Preferred Reported Items for Systematic Reviews and Meta-Analysis (PRISMA) [17]. PubMed, Embase and Cochrane online databases were searched from January 12,000 to October 272,018 to select qualified studies evaluating the diagnosis accuracy of mpMRI for the detection of prostate cancer. The search string combined synonyms of prostate cancer, MRI as follows: (prostate cancer OR prostatic cancer OR prostate neoplasm OR prostatic neoplasm OR prostate tumor OR prostatic tumor OR prostate (magnetic e resonance imaging OR MRI OR MR) carcinoma OR prostatic carcinoma OR prostate cancer). We included all original studies if they satisfied all the following requirements.

I. Studies should be prospectively designed without subjects-selection bias.
II. The available data is sufficient enough to calculate the diagnostic sensitivity and specificity of mpMRI.
III. The pathology results were provided by prostatectomy or prostate biopsy as reference to verify the mpMRI diagnose.
IV. Sufficient data of at least 10 patients is provided to construct 2 × 2 contingency tables.
V. The enrolled patients underwent T2WI and at least one functional imaging technique such as DWI, DCEI, or magnetic resonance spectroscopic imaging (MRSI). The Quality Assessment of Diagnostic Accuracy Studies 2 (QUADAS-2) tool was used to ascertain the quality of studies and likelihood of bias. The included articles were evaluated from the following aspects to decide if they were eligible for further analysis: index test (Describe the index test and how it was conducted and interpreted), patient selection (Describe methods of patient selection), flow and timing (Describe the interval and any interventions between index tests and the reference standard), reference standard (Describe the reference standard and how it was conducted and interpreted), as well as the concerns for applicability. Studies with high risk bias in more than two indexes would be excluded from our research. Only the studies involving biopsy-naive patients and/or patients with a history of the negative biopsy were adopted, and there was no restriction on the biopsy technique or the number of biopsies.

Two reviewers (LZ and YY) independently checked titles and abstracts of all retrieved articles and determined final eligibility according to the previously mentioned criteria. Any disagreements between reviewers required consensus or references of a third reviewer (LX). All screening was performed by a pre-specified data extraction form.

The meta-analysis was carried out through a random effects model in RevMan 5.3. Measures such as diagnostic accuracy, including area under the receiver operating characteristic curve (AUC), sensitivity, specificity, positive predictive value and negative predictive value were calculated with corresponding 95% CIs [18]. Data were analyzed with Review Manager 5 and Meta-Disc.

Multivariate meta-regression for sensitivity and specificity were applied to explore the possible source of heterogeneity. The factors that may have an impact on performance of mpMRI such as patient enrollment (consecutive versus not consecutive), reference standard (high risk of bias versus low risk of bias), whether the readers were blinded to histologic findings (blinded versus not blinded), the application of endorectal coil (ERC) (applied versus not applied), MRI field strength (3 T versus 1.5 T), whether DCEI was performed (mutiparametric versus biparametric) were introduced as variables into the meta-regression where *P* < 0.05 indicated a contribution to heterogeneity. The subgroup meta-regression was based on data from the main analysis.

Heterogeneity was assessed using the I^2^ statistical method, with I^2^ > 50% or *P* value  <  0.05 indicating significant heterogeneity. Deeks’ analysis was performed to evaluate the publication bias, with *P* < 0.05 suggesting publication bias [19].

## Results

### Study selection

**Fig. 1:**
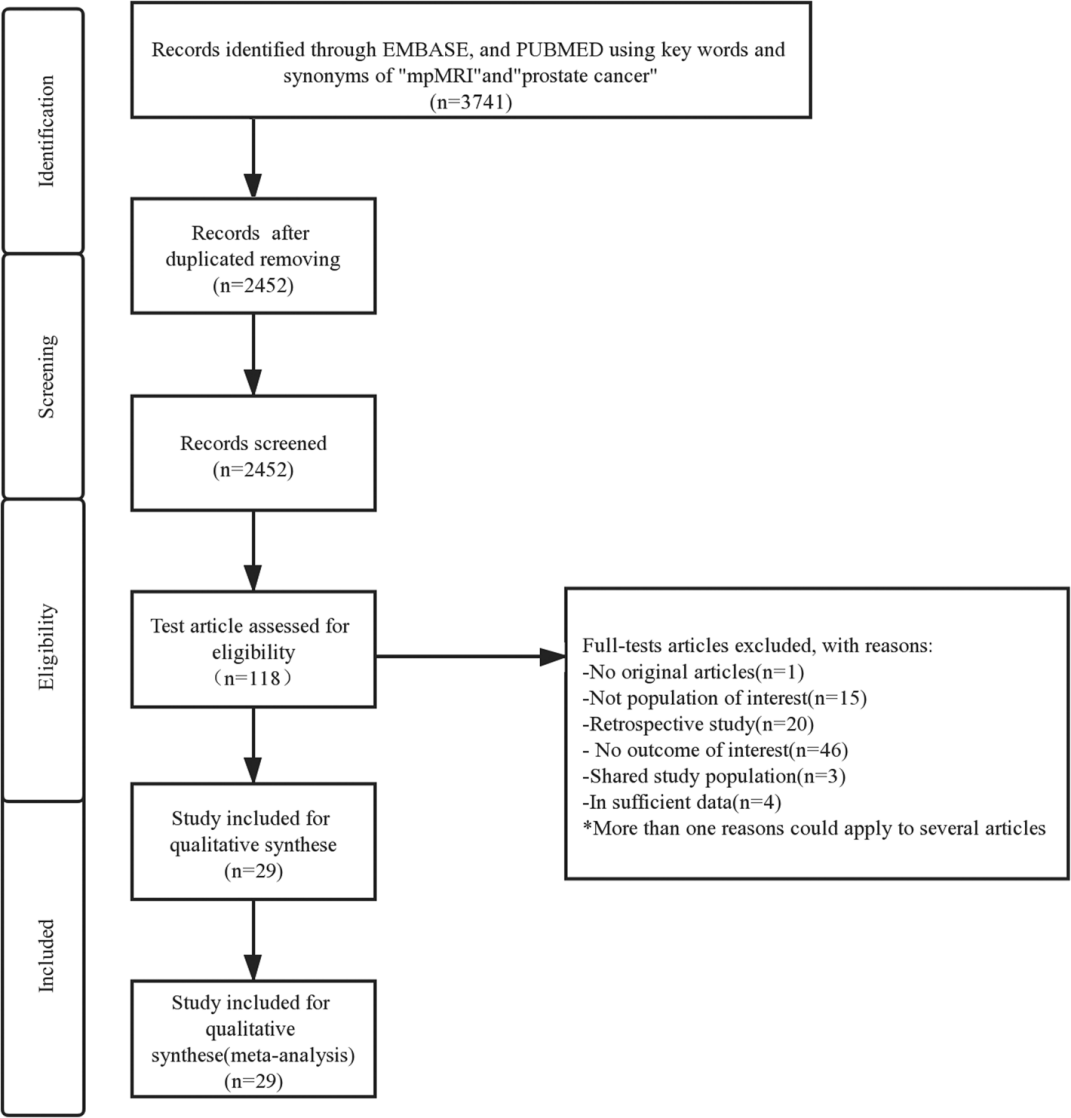
Preferred reporting items for systematic reviews and Meta-analysis flow chart

The study selection process and reasons for exclusion were depicted in the flow diagram [Fig. 1]. A total number of 3741 citations were initially identified. After abstract screening and removal of duplicates, 325 studies were selected for detailed evaluation. At last, 29 studies met all eligibility criteria [3, 15, 20–46].

### Characteristics of included studies

Eight thousand five hundred three who underwent prostate pre-biopsy mpMRI were involved in the 29 studies. The population size of study varied from 26 to 1448 patients. The involved 29 cohorts were carried out in the China, Egypt, Romania, Switzerland, Australia, France, USA, UK, Japan, Germany, Italy and India. The publication time ranged from 2007 to 2017. The study and patient elementary characteristics were displayed in Table 1. The target patients were biopsy-naive men in 8 studies [3, 15, 20, 22, 23, 37, 38, 41], and men with at least one previous negative biopsy in 8 studies [21, 25, 31, 34, 39, 42, 44, 45]; in 9 studies [26, 29, 30, 32, 33, 35, 40, 43, 46] the biopsy history of the patients was unclear. In rest studies patients were both biopsy-naive men and men with a history of previous negative biopsy. The age range of men was from 26 to 91 years (with an average of 65.8) and the PSA value ranged from 0.02–9796 ng/ml.

**Table 1:** Baseline characteristics of included studies

Author	Period	Pt Nb	Repeat setting	Mean/median age (yr)	Age range (ng/ml)	Mean/median PSA	PSA range	Mean/median prostate volume (cm)	Prostate volume range	MR pulse sequences	Magnetic field	Endorectal coil	Classification criteria	Reference
Jagannathan(2017) [20]	2016	26	FB	65.9	37–86	NR	NR	NR	16–144	T2WI/DWI/MRSI	3	Y	NR	sbx
Popita(2017) [21]	2013–2016	39	PNB	68.02	51–78	22.69	median = 12.95	NR	NR	T2WI/DWI/DCEI	1.5	Y	PIRADS	sbx
Gaunay(2017) [22]	NR	400	FB	NR	NR	NR	NR	NR	NR	T2WI/DWI/DCEI	3	NR	PIRADS	rp
Wang(2016) [23]	2002–2009	586	FB	70	26–91	11.11	0.02–9796	NR	NR	T2WI/DWI/DCE/MRSI	3	NR	PIRADS	sbx
Brock(2015) [24]	2013–2014	168	FB & PNB	64	59–70(IQR)	9.2	6.7–13.4(IQR)	55.4	42–80	T2WI/DWI/DCEI	1.5	Y	PIRADS	sbx
Hauth(2015) [25]	2011–2013	94	PNB	64	43–83	9	3~31	51	17–140	T2WI/DWI/DCE/MRSI	3	N	PIRADS	sbx + tbx
Panebianco(2015) [26]	2011–2014	925	NR	NR	NR	NR	NR	NR	NR	T2WI/DWI/DCEI	1.5	N	PIRADS	sbx + tbx
Pepe(2015) [15]	2011–2014	100	FB	64	NR	8.6	4.2–10	NR	NR	T2WI/DWI/DCEI/MRSI	1.5	Y	PIRADS	tpbx
Radtke(2015) [27]	2013	294	FB (63.3%) &PNB (36.7%)	64	61–70	7.3	SD = 6	47	NR	T2WI/DWI/DCEI	3	N	PIRADS	sbx + tbx
Thompson(2014) [28]	2012–2013	150	FB (88%) &PNB (12%)	62.4	55–66.4(IQR)	5.6	4.5–7.5(IQR)	40	30–57	T2WI/DWI/DCEI	3	N	PIRADS	tpbx
Alazeez(2014) [29]	2007–2011	129	NR	62	41–82	5.8	1.2–20	40	16–137	T2WI/DWI/DCEI	3	Y	Dichotomous	sbx
Javali(2014) [30]	2002–2011	140	NR	Control:62.4 Study:62.9	NR	Control: 6.8 Study: 6.87	NR	Control:44 Study: 43	NR	T2WI/MRSI	1.5	N	1–5 scale (Likert)	sbx
Matsuoka(2014) [31]	2007–2012	135	PNB	67	50–80	7	2.9–19.8	25.4	12.7–90.2	T2WI/DWI	3	N	PIRADS	tbx
Petrillo(2014) [32]	2009–2010	136	NR	NR	NR	NR	NR	NR	NR	T2WI/DWI/MRSI	1.5	Y	1–5 scale (Likert)	sbx
Pokorny(2014) [3]	2012–2013	223	FB	63	57–68	5.3	4.1–6.6	41	NR	T2WI/DWI/DCEI	3	N	PIRADS	sbx + tbx
Porpiglia(2014) [33]	2011–2013	170	NR	65	60–70	6.9	5.2–9.8	42	36–48	T2WI/DWI/DCEI	1.5	Y	PIRADS	sbx
Busetto(2013) [34]	2010–2011	163	PNB	66.4	NR	6.8	NR	NR	NR	T2WI/DWI/DCEI/MRSI	3	Y	NR	sbx + tbx
Ferda(2013) [35]	NR	164	NR	NR	49–74	NR	4.2–123	NR	NR	T2WI/DWI/DCEI/MRSI	3	N	NR	sbx
Kuru(2013) [36]	2010–2011	347	FB (51%) &PNB (49%)	65.3	42–82	9.85	0.5–104	48.7	9–180	T2WI/DWI/DCEI	3	N	1–3scale(Likert)	sbx + tbx
Numao(2013) [37]	2006–2010	351	FB	65	59–70	6.3	4.9–9.1	32	24–42	T2WI/DWI/DCEI	1.5	N	1–5 scale (Likert)	tpbx
Ibrahiem(2012) [38]	2008–2009	100	FB	65.03	SD = 7.13	26.3	SD = 24.2	60/09	NR	T2WI/DWI	1.5	N	Dichotomous	sbx
Portalez(2012) [39]	2011	129	PNB	64.7	47–79	9.6	2.7–40	51.1	12–192	T2WI/DWI/DCEI	1.5	NR	1–5 scale (Likert)PI-RADS	sbx + tbx
Watanabe(2012) [40]	2004–2008	1448	NR	72	SD = 7.5	NR	NR	NR	NR	T2WI/DWI	1.5	N	NR	sbx + tbx
Haffner(2011) [41]	2005–2008	555	FB	64	47–83	6.75	0.18–100	46	15–200	T2WI/DCEI	1.5	N	1–5 scale (Likert)	sbx + tbx
Rouse(2011) [42]	2005–2007	114	PNB	63.6	41–83	13.4	0–228	NR	NR	T2WI/DWI/DCEI	1.5	NR	1–5 scale (Likert)	sbx
Kitajima(2010) [43]	2008–2009	53	NR	69	56–84	11.1	4.2–112.1	NR	NR	T2WI/DWI/DCEI	3	N	1–5 scale (Likert)	sbx
Labanaris(2010) [44]	2004–2008	260	PNB	NR	NR	NR	NR	NR	NR	T2WI/DWI/DCEI	3	Y	Dichotomous	sbx
Sciarra(2010) [45]	2007–2009	110	PNB	63.5	49–74	NR	NR	NR	NR	T2WI/DCEI/MRSI	1.5	N	NR	sbx + tbx
Kumar(2007) [46]	NR	61	NR	65.3	SD = 9.3	16.5	0.21–155	NR	NR	T2WI/MRSI	1.5	N	NR	sbx + tbx

The magnetic field strength was 1.5 T in 17 studies [20, 21, 23, 25, 26, 30–32, 37–42, 44, 46, 47] and 3 T in 10 studies [3, 15, 22, 24, 27, 34–36, 43, 45], respectively. DWI was conducted in 26 studies [3, 15, 20–29, 31–40, 42–44] and DCEI was conducted in 20 studies [3, 15, 21–29, 33–37, 39, 42–44], respectively. 10 studies [15, 20, 23, 25, 30, 32, 34, 35, 45, 46] also adopted MRSI. An ERC was used in 9 studies [15, 20, 21, 24, 29, 32–34, 44]. The definition of positive mpMRI was different from studies. Prostate Imaging Reporting Data System (PI-RADS) score system was used in 11 studies [3, 15, 21–28, 31, 33]. The reference standard was based on radical prostatectomy (RP) in 1 studies [22], TRUS-guided systematic biopsy in 22 studies [3, 20, 21, 23–27, 29, 30, 32–36, 38–44, 46], MRI-TRUS fusion–guided targeted biopsy or MRI-guided biopsy in 12 studies [3, 25–27, 31, 34, 36, 39–41, 45, 46]., transperineal template saturation biopsy in 3 studies [15, 28, 37]. Some studies used the combination of these standards. The per-patient analysis was adopted in 24 studies [3, 20–22, 24–29, 31–38, 40, 41, 43–46], others were analyzed by lesion or lobe.

### Sensitivity and specificity of pre-biopsy mpMRI

**Fig. 2:**
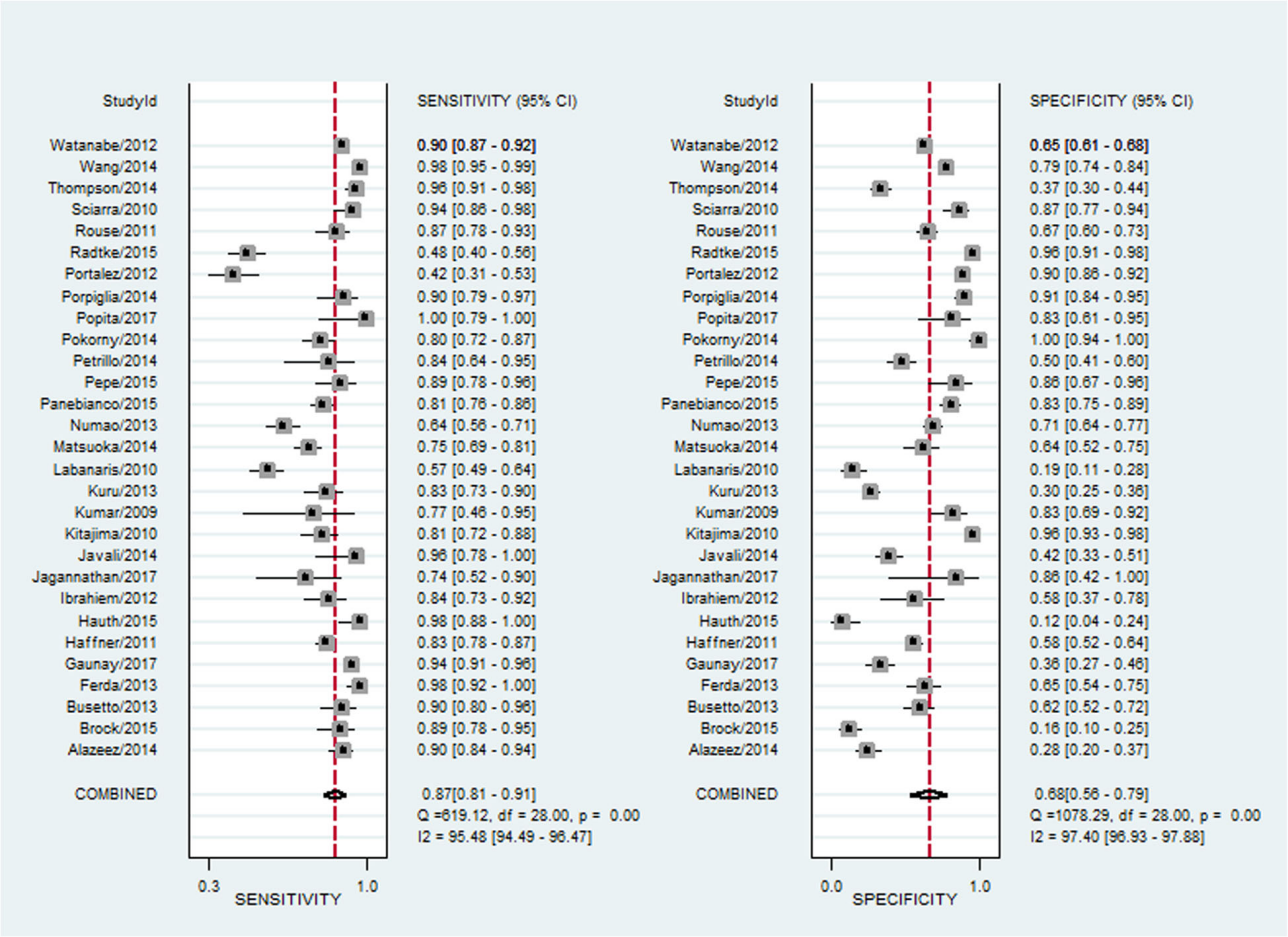
Coupled forest plots of pooled sensitivity and specificity. Numbers are pooled estimates with 95% CI in parentheses. Corresponding heterogeneity statistics are provided at bottom right corners

**Fig. 3:**
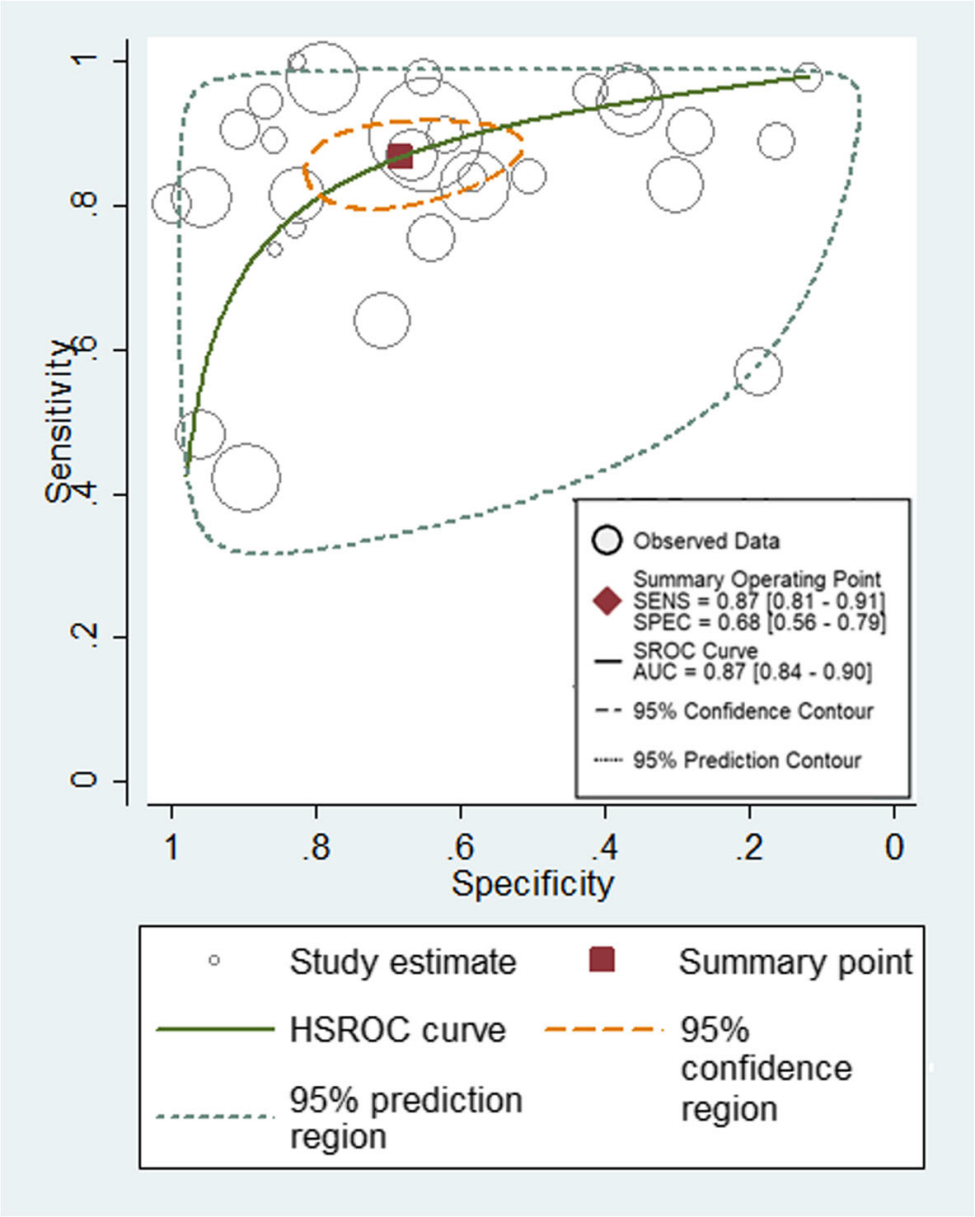
Hierarchical summary receiver operating characteristic curve of the diagnostic performance of mpMRI for detecting prostate cancer

The diagnostic performance of prebiopsy multiparametric MRI of each included studies were demonstrated in Table 2. The sensitivity of mpMRI ranged from 42 to 100%, and the specificity ranged from 12 to 100%. The pooled sensitivity was 0.87 [95% CI (0.81–0.91)] with heterogeneity (I^2^ = 95.48 *P* < 0.01) and a pooled specificity of 0.68 [95% CI (0.56–0.79)] with heterogeneity (I^2^ = 97.40, *P* < 0.01) [Fig. 2]. At the patient level, the median biopsy positive rate was 49%. The area under the hierarchical summary receiver operating characteristic curve (HSROC) was 0.87 (95%CI, 0.84–0.90) [Fig. 3]; positive likelihood ratio was 2.73 [95%CI 1.90–3.90]; negative likelihood ratio was 0.20 (95%CI, 0.14,-0.27); and diagnostic odds ratio (DOR) was 14.00 (95%CI, 7.88–24.84). The median mpMRI NPV was 0.79 (IQR, 0.70–0.92).

**Table 2:** Diagnostic performance of prebiopsy multiparametric MRI using biopsy findings as reference

Author	Prevalence	Reporting Level	Tn	Fn	Tp	Fp	Sensitivity	Specificity	NPV
Jagannathan(2017)	0.73	Patient	6	6	17	1	0.89	0.86	0.50
Popita(2017)	0.57	Patient	19	0	16	4	1.00	0.83	1.00
Gaunry(2016)	0.48	Patient	41	24	376	72	0.94	0.36	0.63
Brock(2015)	0.58	Patient	17	7	56	88	0.89	0.16	0.71
Hauth(2015)	0.42	Patient	6	1	42	45	0.98	0.12	0.86
Panebianco(2015)	0.45	Patient	104	43	186	22	0.81	0.83	0.71
Pepe(2015)	0.54	Lobe	24	6	49	4	0.89	0.86	0.80
Radtke(2015)	0.51	Patient	138	78	72	6	0.48	0.96	0.63
Thompson(2015)	0.61	Patient	74	6	137	127	0.96	0.37	0.92
Wang(2015)	1.00	Patient	200	8	332	53	0.98	0.79	0.96
Alazeez(2014)	0.16	Patient	33	14	127	84	0.90	0.28	0.70
Javali(2014)	0.64	Lobe	49	1	22	68	0.96	0.42	0.98
Matsuoka(2014)	0.37	Patient	46	49	149	26	0.75	0.64	0.48
Petrillo(2014)	0.18	Patient	56	4	21	55	0.84	0.50	0.93
Pokorny(2014)	0.63	Patient	56	25	101	0	0.80	1.00	0.69
Porpiglia(2014)	0.30	Patient	107	5	47	11	0.90	0.91	0.95
Busetto(2013)	0.41	Patient	59	7	61	36	0.90	0.62	0.89
Ferda(2013)	0.51	Patient	52	2	82	28	0.98	0.65	0.96
Kuru(2013)	0.57	Patient	80	14	67	186	0.83	0.30	0.85
Numao(2013)	0.45	Patient	136	57	101	56	0.64	0.71	0.70
Ibrahiem(2012)	0.73	Patient	14	11	57	10	0.84	0.58	0.56
Portalez(2012)	0.48	Lesion	404	47	34	47	0.42	0.90	0.89
Watanabe(2012)	0.48	Patient	485	73	624	266	0.90	0.65	0.86
Haffner(2011)	0.54	Patient	154	50	240	111	0.83	0.58	0.75
Rouse(2011)	0.33	Sextant	145	11	74	72	0.87	0.67	0.92
Kitajima(2010)	0.56	Patient	311	19	80	14	0.81	0.96	0.94
Labanaris(2010)	0.73	Patient	17	73	96	74	0.57	0.19	0.18
Sciarra(2010)	0.34	Patient	61	4	66	9	0.94	0.87	0.93
Kumar(2009)	0.21	Patient	39	3	10	8	0.77	0.83	0.92

### Subgroup analysis

According to our subgroup analysis, many factors, such as patient enrollment (consecutive versus not consecutive), reference standard (high risk of bias versus low risk of bias), whether the readers were blinded to histologic findings (blinded versus not blinded), the application of ERC, and MRI field strength (3 T versus 1.5 T) showed significant independence associated with sensitivity (*P* < 0.05 for all). Moreover, these previously mentioned factors affected only sensitivity, and none of those factors has an impact on specificity. As for the subgroup exploring the application of DCEI, there is no significant difference between two groups in both sensitivity and specificity. The results of our subgroup analysis were shown in Table 3.

**Table 3:** Subgroup analysis of sensitivity and specificity according to the mpMRI

parameter	category	nstudies	sensitivity	P1	specificity	P2
coil	used	9	0.89 (0.83–0.89)	<0.05	0.67 (0.46–0.88)	0.69
	Not used	16	0.86 (0.80–0.92)		0.66 (0.50–0.83)	
magnetic	3	10	0.88 (0.81–0.95)	<0.05	0.77 (0.61–0.93)	0.99
	1.5	17	0.85 (0.78–0.91)		0.67 (0.51–0.82)	
reference	PI-RADS	11	0.90 (0.84–0.95)	0.17	0.60 (0.37–0.83)	0.17
	others	9	0.83 (0.74–0.92)		0.78 (0.61–0.96)	
MRI	mutiparametric	22	0.88 (0.83–0.93)	0.19	0.72 (0.58–0.85)	0.57
	biparametric	7	0.82 (0.70–0.93)		0.56 (0.30–0.82)	
enrollment	consecutive	22	0.85 (0.80–0.91)	<0.05	0.71 (0.58–0.84)	0.77
	Not consecutive	7	0.91 (0.84–0.98)		0.59 (0.33–0.85)	
blinding	blinded	14	0.83 (0.75–0.90)	<0.05	0.73 (0.58–0.88)	0.97
	Not mention	15	0.90 (0.85–0.95)		0.63 (0.45–0.80)	
standard	Low bias	12	0.84 (0.75–0.92)	<0.05	0.74 (0.58–0.90)	0.96
	High bias	17	0.88 (0.83–0.94)		0.64 (0.48–0.90)	
Biopsy naive	Yes	9	0.87 (0.80–0.95)	0.35	0.64 (0.40–0.88)	0.28
	No	7	0.81 (0.70–0.92)		0.78 (0.59–0.97)	

### Quality of studies

**Fig. 4:**
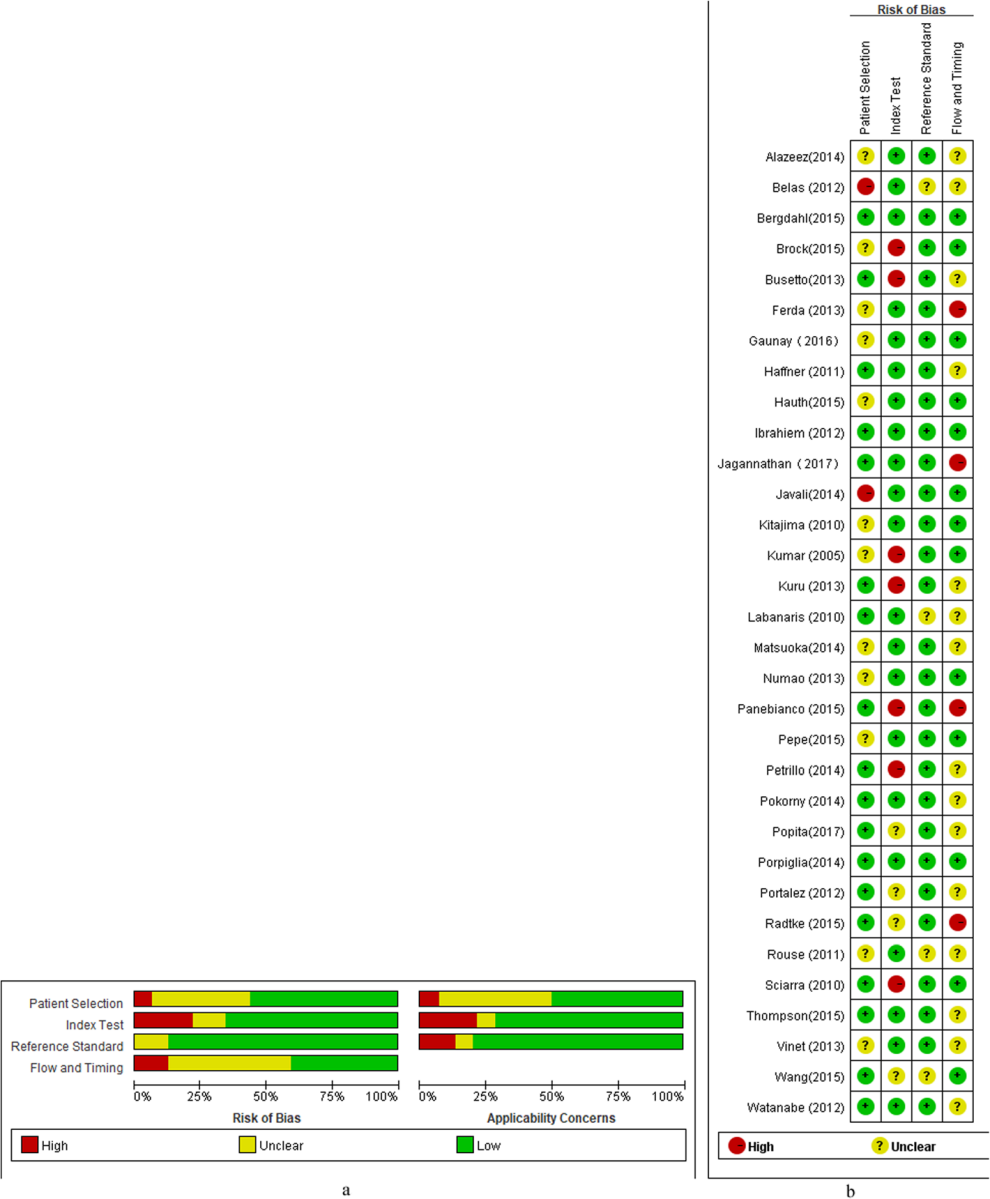
(**a**)- Assessment of the risk of bias for included studies. (**b**)- Risk of bias summary graph

**Fig. 5:**
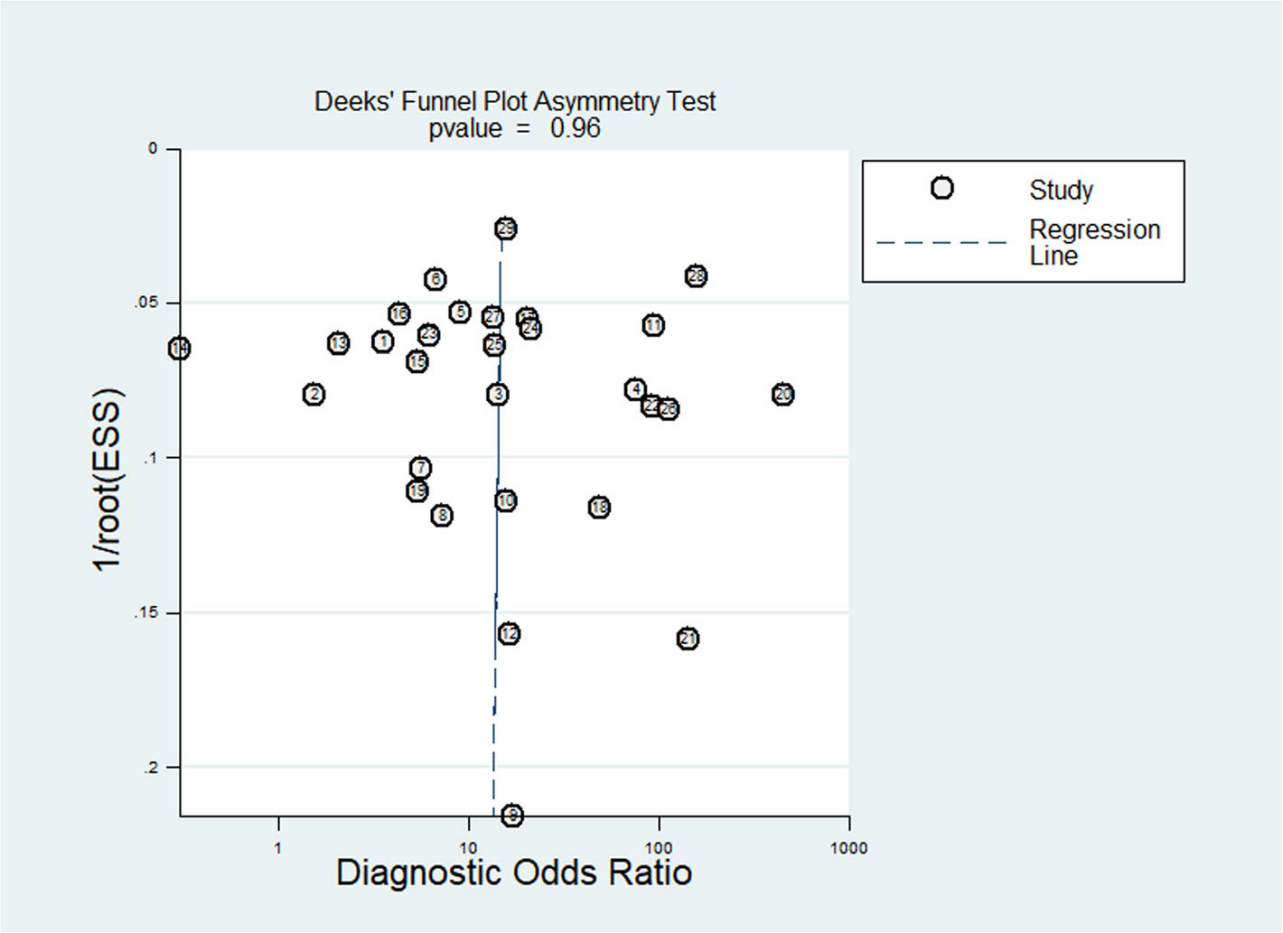
Deeks’ funnel plot. A *p* value of 0.961 suggests that the likelihood of publication bias is low.

Regarding the patient selection domain, 7 studies [15, 22, 29–31, 35, 46] had a high risk of bias because consecutive enrollment was not used or did not mention the exclusion criteria. Regarding the index test domain, in 10 studies [15, 25, 26, 30, 32–35, 44, 46] the cut-off value for determining PCa was not specifically prior to interpretation. 6 studies [30, 31, 38, 40, 41, 46] did not have complete MRI parameters. Regarding the reference standard domain, we considered RP and targeted biopsy as the low risk reference standard. The TRUS-guided systematic biopsy or transperineal saturartion biopsy were considered to have a high risk of bias. Therefore, the risk of bias regarding the reference standard was high in 12 studies [3, 15, 24–27, 33, 34, 38, 40, 41, 45]. In 13 included studies, [3, 15, 27–29, 31, 32, 37, 38, 42, 44, 45, 47] the blinding method was applied; however, other 16 studies did not explicitly mention blind method. There was no high risk of bias in any of the included studies in flow and timing domain [Fig. 4a and b]. The Higgins I^2^ statistics illustrated remarkable heterogeneity in terms of the sensitivity (I^2^ = 95.48%) and specificity (I^2^ = 97.40%). According to the Deeks’ funnel plot, the likelihood of publication bias was low, with a *p* value of 0.96 for the slope coefficient.[Fig. 5].

## Discussion

In our meta-analysis, we assessed the diagnostic accuracy of mpMRI for detecting PCa. The results of our meta-analysis showed that the diagnostic accuracy of mpMRI for detecting PCa in 29 included studies was high with a sensitivity of 0.87 and a specificity of 0.68 respectively.

Compared with the former review, the current study is the first meta-analysis to evaluate the performance of mpMRI based on prospective studies which could minimize the selection bias, besides, multiple subgroup analysis were performed in our study to explore the potential factors that may have an impact on the accuracy of mpMRI. Based on our results, pooled sensitivity was significantly higher in coil application group than in group without coils. Furthermore, the comparison between different magnetic strength suggested that field strength of MRI may also influence the accuracy of the diagnostic trial, the sensitivity from 3 T group is significantly higher than that from 1.5 T group. On the contrary, the comparison between biparametric MRI (bpMRI) (mpMRI without DCEI) and mutiparametric MRI (mpMRI with DCEI) demonstrated similar point estimates for sensitivity and specificity. Through our subgroup analysis, the caregivers could offer the most suitable method of mpMRI for the pre-biopsy patients with suspected prostate cancer.

As the application of 3 T MRI became increasingly popular, mpMRI played an important role in the diagnostic of PCa. Murphy et al. [48] reported that increasing the magnetic field strength could improve the diagnostic accuracy of PCa. The study of Sertdemir et al. [49] suggested that prostate cancer could be better identified from prostatitis at 3 T strength field compared with 1.5 through DCEI. Theoretically, the MRI signal strength is proportional to the square of the static field strength while imaging noise increases drastically [50]. Barth et al. [51] indicated that the signal-noise ratio (SNR) of 3 T scanners is twice as good as 1.5 T in optimal condition. Several studies have also demonstrated the advantages of higher field strengths. However, the disadvantages and new challenges accompanied by higher field strength such as increased power deposition, artifacts related to susceptibility, and signal heterogeneity also should not be ignored. Methods to prevent those effects still require further exploration [52]. In addition, Nieuwenhove et al. [53] indicated that a fast MRI protocol (1.5 T magnet, T2 + DWI, < 15 min) may replace the traditional 3.0 T mpMRI protocol safely without missing clinical significance so that the cost and contrast injection could be saved. Nevertheless, its conclusion was limited by a small number of endpoints. To validate these conclusions, a larger multicenter population-based trial is needed .

Although ERC has been applied to clinical practice since the early 1990s, its results have not been very heartening until the emergence of mpMR [54]. During the MRI examination, the ERC is inserted into rectal and clung tightly to the prostate to improve the image resolution and staging accuracy [55]. The application of the ERC may lead to higher costs because of material and longer examination time [56]. Moreover, many patients experienced discomfort feelings during the use of an ERC as well as the ensuing examination [57]. These disadvantages might limit the acceptance of ERC in mpMRI process. How to shorten the examination time and decrease the costs of imaging protocol are the problems researchers need to consider. Data of the study showed that ERC acceptance was directly related to how comfortable patients were. However, if such an examination was considered necessary, most patients would have been willing to undergo another examination with ERC and only 30% of patients will suffer from impaired diagnostic accuracy as a trade-off for higher patient comfort with the absence of ERC [58].

Limitations of mpMRI as an adjunct tool for prostate cancer screening are also noteworthy, such as increasing acquisition time, and the safety issue of gadolinium [59]. Recent studies have demonstrated high accuracy of bpMRI [60, 61]. The analysis of the subgroup showed that bpMRI did not differ significantly from that of mpMRI which included an additional DCEI-MRI sequence. (Table 3) Currently, whether to include DCEI-MRI in prostate MRI is a timely and controversial subject. Many articles focusing on bpMRI or comparisons between bpMRI and mpMRI were published [62, 63], and sessions focusing on this subject were being held at international congresses (i.e., European Association of Urology 2017, Korean Congress of Radiology 2017, and European Congress of Radiology 2018). In a study published in 2017, Greer et al. [64] suggested that the application of DCEI-MRI could benefit the diagnostic of prostate cancer because abnormal DCEI-MRI findings improve the cancer detection rate in each of the PI-RADSv2 categories 2, 3, 4, and 5. However, those who advocated the use of bpMRI or opposed the use of DCEI-MRI suggested that compared with mpMRI, bpMRI has several advantages such as shorter examination time, avoidance of risks associated with gadolinium-based contrast agents, and minimal risk of missing csPCa. Vargas et al. [65] found that DCEI-MRI helped to find only four additional tumors out of 152 patients. In addition, Kuhl et al. [61] demonstrated that the application of bpMRI protocol could reduce the MRI acquisition time from 34 min 19 s to 8 min 45 s. However, most of the studies advocating bpMRI were retrospective studies with small number of patients. Larger- scale trials comparing bpMRI and mpMRI for further study was needed.

Our study also indicated that there was no statistically significance in both sensitivity and specificity between studies applied PI-RADS and studies with other criteria.The insignificance may owe to that multiple studies in PI-RADS group administrated PI-RADS version one as criteria. When we separated studies based on PI-RADS v2 from studies [21, 23, 25, 35] on old version and performed the meta-regression again, both sensitivity and specificity of PI-RADSv2 group were significantly higher than that from other group. The sensitivity of PI-RADSv2 group was 0.89 (0.81–0.97) and others was 0.88 (0.82–0.93) with *p* = 0.04.

In order to standardize the acquisition, interpretation and reporting of prostate mpMRI, the European Society of Urogenital Radiology proposed the PI-RADS in 2012 [66]. In December 2014, the updated and simplified version (PI-RADSv2) was introduced to address the limitations and issues derived from the old version [67]. It summarized the level of likelihood of PCa in a five-point scale based on mpMRI findings considering the combination of T2WI, DWI, and DCE. One previous meta-analysis made a direct comparison between the two versions indicating that updated PI-RADSv2 showed significant improvement compared with the original PI-RADSv1 [68]. A more recent study which retrospectively investigated 166 patients after RP suggested PI-RADS v2 could be used to predict long-term outcomes following RP. PI-RADS could diminish variation in the interpretation and reporting of prostate imaging, especially among readers with varied experience levels [69]. Multiple studies has also proved mpMRI based on PI-RADS was useful in preventing unnecessary invasive procedures and helpful for the prediction and diagnosis of PCa and csPCa when combined with PSA and PSAD [70].

The administration of mpMRI for the evaluation of prostate cancer has increased drastically and this trend is likely to keep going because the technology is rapidly improving and its applications are expanding. A great number of previous studies have been focused on the accuracy of negative pre-biopsy prostate mpMRI in predicting a negative biopsy result for csPCa. However, the huge difference in csPCa definition prevented any explicit conclusions about mpMRI’s ability to rule out aggressive cancer. Since biopsy results could not accurately reflect tumor burden and aggressiveness, how to define csPCa on biopsy appropriately is a complicated issue. Therefore, it is an urgent need to standardize the histological definition of csPCa in order to make comparisons among studies more meaningful.

The basic characteristic of imaging biomarkers is reproducibility as any biomarker fails its purpose, if it could not be reproducible or transfer to other patients, scanners or imaging protocols. Inter-observer variability is the reflection of reproducibility and is defined as the systematic differences among different observers [71]. Numerous acquisition techniques have been proposed in the past to solve the inter-observer variability in prostate cancer MRI. The occurence of quantitative MRI analysis has made it possible to compare between patients from different centers through quantitative hemodynamic parameters, and explore the change of biological characteristics before and after treatment. Instead of visualizing tissue characteristics indirectly through weighted contrasts, quantitative MRI attempts to directly measure them. Quantitative measurement of apparent diffusion coefficient (ADC) values has shown impressive diagnostic performance in discriminating csPCa from insignificant PCa, with overall AUC 0.880, and sensitivity of 71% and specificity of 88% and it has been proved these results are reproductive with high inter-observer correlation among different radiologists [72]. Although DCE played a minor role in the diagnosis of PCa, it could describe the suspicious lesions in quantitative and semi-quantitative measures which were different from DWI and T2 W1. Daniel et al. [73] indicated that combined with individualized T1-time correction, DCE could achieve excellent reproducibility, both intra- and inter-observer variability were found to be increased.

Magnetic resonance fingerprinting (MRF) is a newly invented nuclear magnetic resonance parameter mapping method proposed by Ma et al. [74] which could estimate several quantitative tissue property parameters like T 1 and T 2 relaxation times simultaneously through transient signal evolutions and data analysis. With this ability, MRF could provide a solution to the problem of obtaining quantitative measures in an efficient manner and in short scanning times. Previous studies have demonstrated high reproducibility of MR fingerprinting parameter maps in solid tissues in the supratentorial region of the human brain [75]. Whether it could also improve the reproducibility and repeatability in PCa detection still requires the support of larger multicenter, randomized control trials. Moreover, 3D 1H-MRSI of the prostate has also been proved by Lagemaat et al. [76] as an reproducible technique, however that conclusion was drawn based on small sample which limited its reliability; the larger population-based studies are also needed to prove that conclusion in the future.

## Limitations

I. Included studies were heterogeneous in their methods, which may have an impact on the general applicability of the summary results. On the other hand, the methodologic variability can provide a lot of information for our subgroup analysis so that we can improve the diagnostic accuracy of mpMRI by identifying those factors in the future. II. Until recently, the definition of clinically relevant PCa varied considerably between each study. Therefore, we did not explore the possibility whether the prostate biopsy could be avoided through pre-biopsy MRI. III. Patients considered to be positive (signs and symptoms of prostate cancer) are more likely to get the gold standard, and only patients who get the gold standard are included in the study. This would result in falsely decreasing specificity and increasing sensitivity. It highlighted further areas of research that could help in defining the best use of mpMRI in the early detection of aggressive prostate cancer in the future. IV. Finally, data in some included studies are not provided completely, so we have to calculate the sensitivity and specificity by ourselves, this may affect the overall result of our study to some extent.

## Conclusion

Although mpMRI can detect prostate cancer with excellent sensitivity, four main issues must be addressed before it becomes a triage test of prostate biopsy. I. The definition of the csPCa must be explicit so that further studies can be carried out to select the patients, the biopsy of whom can be obviated. II. The coil, the mpMRI with a stronger magnetic field, were recommended to improve the diagnostic accuracy of prostate cancer. III. The application of DCEI in the diagnostic of prostate cancer still needs to be testified. IV. Although efforts to standardize mpMRI technical protocols and interpretation have been made over the past few years, it is still urgent to improve mpMRI specificity and inter-reader reproducibility.

## Data Availability

All data is included in the article and additional fles.

## Abbreviations

bpMRI: Biparametric MRI
Bx: Biopsy
csPCa: Clinically significant cancer
DCEI: Dynamic contrast-enhanced imaging
DOR: Diagnostic odds ratio
DWI: Diffusion-weighted imaging
ERC: Endorectal coil
FB: First biopsy
FN: False negative
FP: False positive
HSROC: Hierarchical summary receiver operating characteristic curve
IQR: Interquartile range
mpMRI: Multiparametric magnetic resonance imaging
MRSI: Magnetic resonance spectroscopic imaging
NPV: Negative predictive value
NR: Not reported
PCa: Prostate cancer
PNB: Previous negative biopsy
PPV: Positive predictive value
RoB: Risk of bias
ROC: Receiver operating characteristic curve
RP: Radical prostatectomy
SNR: Signal-noise ratio
T2: Weighted imaging
TBx: Targeted biopsy
TN: True negative
TP: True positive
TPBx: Transperineal template biopsy
TRUS SBx: Transrectal ultrasound-guided standard biopsy
